# Using already-solved cases of a mass disaster event for prioritizing the search among remaining victims: a Bayesian approach

**DOI:** 10.1038/s41598-020-59841-3

**Published:** 2020-03-19

**Authors:** Inés Caridi, Enrique E. Alvarez, Carlos Somigliana, Mercedes Salado Puerto

**Affiliations:** 1 Instituto del Cálculo and CONICET, Facultad de Ciencias Exactas y Naturales, Universidad de Buenos Aires, Buenos Aires, Argentina; 2 Facultad de Ingeniería, Universidad Nacional de La Plata and CONICET, Buenos Aires, Argentina; 3 Equipo Argentino de Antropologia Forense, Buenos Aires, Argentina

**Keywords:** Applied mathematics, Statistics

## Abstract

This work presents a new method for assisting in the identification process of missing persons in several contexts, such as enforced disappearances. We apply a Bayesian technique to incorporate non-genetic variables in the construction of prior information. In that way, we can learn from the already-solved cases of a particular mass event of death, and use that information to guide the search among remaining victims. This paper describes a particular application to the proposed method to the identification of human remains of the so-called *disappeared* during the last dictatorship in Argentina, which lasted from 1976 until 1983. Potential applications of the techniques presented hereby, however, are much wider. The central idea of our work is to take advantage of the already-solved cases within a certain event to use the gathered knowledge to assist in the investigation process, enabling the construction of prioritized rankings of victims that could correspond to each certain unidentified human remains.

## Introduction

The process of identification that guides searches in contexts such as disaster victim identification (DVI), missing person identification (MPI), migration and other situations of violence (OSV) requires the collection of background information from different sources (e.g. legal courts documents, testimonies from survivors, witnesses and families of the missing)^1^. The identification process is essential not only for the sake of Justice and for humanitarian reasons^2^ but also to offer answers to victims’ families and friends^3–6^. The process of identification usually includes both, (*i*) the construction of hypotheses of identity from the analysis of such background information that needs to be evaluated at a later stage through genetic evidence, and (ii) the validation of the information gathered from a genetic DNA-led process through the comparison of the ante-mortem and post-mortem information. It is our aim in this paper to describe a general method which could contribute to the investigation process by taking advantage of the already-solved cases of a particular mass death event, to use that elicited knowledge for guiding new searches of related unidentified human remains (UHR). Whenever a pattern does exist within the already-solved cases, the method presented here allows us to make predictions in the identification process of the cases still unsolved, and it also makes it possible to minimize any bias from the researcher. Predictions are understood as the act of prioritizing some individuals over others to be more likely related to certain UHR within the same event. The available information is: (i) information regarding the context of the mass death event, such as date and place in which the event has occurred and the total number of victims, (ii) a database with information of reported victims who are potential candidates to correspond with a set of UHR. That database also includes non-genetic variables amenable to be modeled mathematically in the search for patterns, and (iii) information of the set of already-solved cases within the same mass event (i.e. cases already identified). Thus, in essence, we have two lists: the set of potential victims whose human remains have not been found yet (which we denote in the sequel List 1) and the set of UHR which have not yet been identified (which we denote by List 2 in the sequel). The main idea of this work is to update an initial instance of knowledge, in which all possible victims are equally likely to correspond with certain UHR within the same mass death event, into a new instance of knowledge, in which some victims are more likely to match certain UHR, in the light of the data resulting from the already-solved cases. We accomplish that updating process mathematically with the aid of Bayesian techniques. Those are used in two ways: i) to elicit background information about the probabilities of matches from UHRs to potential victims in the form of a prior distribution, and ii) to update that prior into a posterior distribution. In this work, we opt to evaluate the results of our method by using cross-validation techniques, for which we introduce two measures of goodness of fit, that we call *Discriminating Power* (*DP*), and *Efficacy Rate* (*E*), as defined in Section *Validation techniques* below. Basically, the *DP* measures the ability of a model fitted only from a *training sample* to update the probabilities of matches using a *validation sample* (e.g., fresh cases). In turn, the *E* measures how *informative* the model, based on the training sample, results for the validation sample. As outputs, the model firstly identifies the best set of informative non-genetic variables to learn from the already-solved cases of a mass event and provides the optimal way of partitioning those variables. Each of those partitions entails a candidate model that will be evaluated through cross-validation. Secondly, the model gives an analytical expression that, when applied to the best partition of the set of non-genetic variables, which implies a partition of the victims (List 1) in subsets, generates probabilistic scores of victims for unidentified human remains of related cases. It is noteworthy that scores are a function of the data and then, each new piece of information (from both the victims database or the set of already-solved cases) could generate new results.

In the statistical analysis of genetic matched data, the probability of the event “the DNA sample is related to the victim" is usually compared to the alternative “the DNA sample is not related to the victim" by calculating odds ratios. The Bayesian approach updates the quotient of this prior odds (built on non-genetic evidence) to obtain the posterior quotient (posterior odds) by multiplying the prior odds by the likelihood ratio (built on genetic evidence)^7–10^. Very low prior odds and/or low quality and quantity of DNA, both of the material extracted from bones and DNA samples from victims’ relatives are some of the causes which could hinder reaching the required threshold of identification that could be reached by improving biological reference samples. For this reason, it is important to prioritize the possible victims of the event in order to allocate efforts to obtain certain DNA samples from close family donors. A good guide on how to prioritize the research of unsolved-cases would, in principle, allow substantial savings in time and resources. As an example of the use of non-genetic information to prioritize or highlight the searches, the collaborative online platform called “Reuniting Families" uses non-genetic information to flag samples of interest that are manually examined by experts using other available data^11^.

During the last Argentine dictatorship, several circuits of Illegal Detention Centers (IDC) were set up in different locations throughout the country. There, thousands of persons were illegally held without any sort of legal guarantees, tortured and most of them killed. Their unidentified bodies were buried in individual or common graves either within official cemeteries or in clandestine mass graves at military or police compounds. Even today, the fate of most of those disappeared people’s remains is still unknown^1^. Missing people have come to be known as “The Disappeared”. Since 1984 the Argentine Forensic Anthropology Team (EAAF) has been working on the identification of the disappeared using a multidisciplinary approach^12–14^. The identification process related to these events which occurred over 35 years ago presents important challenges. First, the information from reports is incomplete; second, even though there are reference samples available from only approximately half of the victims’ families, in some cases the samples belong to distant relatives with a weaker DNA connection. This is because in many cases, relatives who are very informative from a genetic point of view (e.g., mothers and fathers) have already died or are now very old. Moreover, in some cases, several members of the same family were disappeared, for example, the “missing grandchildren”^5^.

The need to generate hypotheses of identity for the recovered human remains and the fact that in some mass events there are sets of already-solved cases, triggered an interest in developing a model that mathematically systematizes information obtained by already-solved identifications, in such a way that it could be used in the search and generation of new hypotheses of identity for related unsolved cases. A Bayesian model is proposed to learn from already-identified cases and to generate a probabilistic ranking of victims for unidentified related cases. This ranking is, by nature, dynamic, as it can be sequentially updated every time new information from sets is appended (List 1 of victims, List 2 of unidentified remains and the set of solved-cases). In that way, the model allows the identification of the most informative subset of non-genetic variables to detect patterns, which beside achieves results that are significantly better than those obtained just by chance. In other words, once the updating has been formalized, a ranking of suitable victims for the recovered skeletal remains is produced. An important advantage of this method is that it minimizes any bias in the investigation of related cases.

## Results

The methodology presented here was applied to events within the context of the last dictatorship in Argentina, such as the so-called Massacre of Fátima. On August 20th, 1976, ten women and twenty men were killed in the township of Fátima, Province of Buenos Aires, Argentina. Hence, it was a well-delimited event, both geographically and temporally, and it involved a well-known number of people. Until now, 24 out of 30 of them have been identified at different stages. Within all the victims of the dictatorship in Argentina, it is possible to select a subset List 1 of individuals to form the set of candidate victims that could correspond to the unidentified remains List 2 associated with the particular event, such as Fátima.

The first challenge to build scores within the set of eligible victims is to select the non-genetic informative variable or variables to learn from the already-solved cases. With this in mind, a partition within the complete range of non-genetic variables was defined on a grid, which entails a grouping of List 1 into some subsets. Every cell on the grid is associated with a combination of values of those variables.

For simplicity, let us assume for a moment that there were only two variables, of geographical and temporal nature, respectively, such as those related to the place and date of kidnapping of the victims. In that case, boxes on a grid represent GeoTemporal cells in the sequel. For data analysis, narrowing or enlarging the time window widths entails parameters to be calibrated later seeking a balance for an optimal division based on a combination of *DP* and *E*. Thus, every identified individual and every victim are placed in a GeoTemporal cell (subset) based on the date and place of their kidnapping. Then, the main idea is to update the importance of each GeoTemporal cell (GeoTemporal probabilities), which represents the probability that the unidentified human remains of the same event may correspond to a victim belonging to this cell. In the initial instance of knowledge of the problem, GeoTemporal probabilities have to be consistent with what is known before the identifications of the event were made, that is, all the possible victims are equally probable, and therefore those cells with more victims will be more likely. This fact means that the prior probabilities of the cells (before having data from identifications) are proportional to the number of possible victims from every GeoTemporal cell. Moreover, within a given cell, we consider that there are a-priori equal probabilities for each of the individuals belonging to this cell. Updated probabilities of GeoTemporal cells will result from a combination of the prior probabilities and the information of the set of GeoTemporal cells observed from the identified cases of that particular event. Then, for certain unidentified human remains from the same event, a probability score to every possible victim is assigned.

Thus, the first step to formulate the victims rankings scores consists of the construction of an informative probability distribution among possible victims learning about the already-solved cases of the same event, based on non-genetic variables. The second step consists of using those probabilities to prioritize some victims as more likely to correspond with UHR. The last step, left for evaluation by forensic experts, is to physically evaluate the rankings to assist the investigation and to build new hypotheses. The information they provide after this process could be incorporated in a new sequential step of prior elicitation. A tool to implement this feedback from forensic researchers is under construction and will be available in a free and open interface^15^.

### Bayesian framework

In this Section, we introduce the notation to be used in the rest of the paper. Let $S$ represent recovered skeletal or human remains of an individual, although still unidentified (unidentified human remains UHR) associated to a particular mass event of death (this is an element of what we have called List 2 in the *Introduction*). We also denote the set of possible victims that corresponds with $S$ by $V=\{{v}_{1},{v}_{2},\ldots ,{v}_{N}\}$ (this is what we call List $1$ in the Introduction), and denote $\#V=N$ (being $\#$ the total number of elements of the set). Then $P({v}_{i}\ {\rm{is}}\ S)$ denotes the probability that the $i$-th possible victim (${v}_{i}$) corresponds to UHR $S$. Hence the expression $P({v}_{i}\ {\rm{is}}\ \ S)=0$ means that with certainty ${v}_{i}$ is not the UHR $S$, while $P({v}_{i}\ {\rm{is}}\ S)=1$ means that with certainty, ${v}_{i}$ corresponds with $S$.

As mentioned, the purpose of this paper is to update the probability $P({v}_{i}\ {\rm{is}}\ S)$, as computed in an initial instance of knowledge, which ignores any information from the observations of the already-solved cases, into some updated probabilities using information from the already-solved cases. The initial and updated probabilities are called *prior* and *posterior* probabilities, respectively, in Bayesian Statistics. They are denoted mathematically by $P({v}_{i}\ {\rm{is}}\ S)$ and $P({v}_{i}\,{\rm{i}}{\rm{s}}\,S|{\mathscr{D}}$), where the latter is a conditional probability in which the set ${\mathscr{D}}$ denotes the data gathered from the already-solved cases. In our presentation below, we consider that *a priori* there is ignorance concerning the event, in the sense that $P({v}_{i}\ {\rm{is}}\ S)=1$/$n$ is the same value for each of the $i$-th possible victims (a fact modelled by a discrete uniform distribution). However, after the updating process, the posterior probabilities $P({v}_{i}\ {\rm{is}}\ S| {\mathscr{D}})$ differ and lead to a ranking of possible matches to be evaluated by forensic experts. The main idea is to use Bayesian Inference, which explains how to update some prior probabilities after appending new information, to give rise to the posterior probabilities^16^.

In our case, a straightforward application of Bayes’ Theorem entails

1 $$P({v}_{i}\ {\rm{is}}\ S| {\mathscr{D}})=\frac{P({v}_{i}\ {\rm{is}}\ \ S)P({\mathscr{D}}| {v}_{i}\ {\rm{is}}\ S)}{P({\mathscr{D}})},$$

Naturally, apart from the information elicited from the already-solved cases (${\mathscr{D}}$), there could be another type of background information. At this stage, it is worth it to mention that Bayesian inference is now recognized as the most useful model to understand how evidence may be presented logically and impartially in legal proceedings^7^, because the underlying assumptions are all explicit. In our treatment for this manuscript, those assumptions are enumerated as follows.

### Assumptions of the identification problem

The assumptions considered to build probabilities for every individual of set $V$ to correspond to UHR $S$ of a particular mass event of deaths (or simply the *event*) are the following:

- (a) The event is well delimited geographically, temporally (the data of occurrence, called ${T}_{e}$, is known) and in terms of the number of killed individuals (called ${n}_{e}$).
- (b) There is a set of already-solved cases of the event, size ${n}_{s}$, which is a subset of the complete set of deaths (${n}_{s}\ \le \ {n}_{e}$).
- (c) It is known with certainty that recovered UHR $S$ corresponds to one of the set of ${n}_{e}$ individuals related to the event. Moreover, $S$ correspond to just one of the victims, which excludes the possibility of mixed remains.
- (d) The set of possible victims that corresponds to recovered human remains related to the event under study is known.
- (e) The set of already-solved cases (${n}_{s}$) is a random sample from the complete set of deaths of this mass event (${n}_{e}$).
- (f) There is a known set of non-genetic variables associated with each victim (e.g., geographical, political, and time related variables).

From assumptions (a), (c) and (d) it is possible to define the set of victims that correspond to recovered human remains $S$ related to the event, called $V=\left\{{v}_{1},{v}_{2},\ldots {v}_{N}\right\}$ (List $1$, mentioned in the *Introduction*), as the subset of the total set of victims who were kidnapped or missing before date ${T}_{e}$. In this sense, $V$ is defined as the *effective* set of possible victims, because it does not include those victims whose probability is zero. This consideration takes into account only the assumptions enumerated above in this manuscript. In other words, it does not include either the sex or the age of the UHR $S$. Using assumption f), it is possible to associate each victim (both the possible victims and the identified ones in the event) with a non-genetic variable or a set of non-genetic variables, as for example the geographical area, and the period before the date of the event when the kidnapping took place. Given a variable, it is possible to define a *partition* of the set of values of this variable such as a division into blocks or cells of values of the variable. Every cell could be associated to a range of values of the variable, both for numeric or categorical variables. If two variables are considered, each cell could be associated with a pair of values; if three variables are considered, each cell could be associated with a third one, and so on. Once a partition is constructed in the form of cells, it implies a grouping of the set of possible victims in the given subsets.

#### GeoTemporal cells

For the sake of simplicity, the following sections will describe the statistical calculations applied to a set of variables associated with the place and date of the kidnapping of the victims. For this reason, the cells are called “*GeoTemporal*”. The number of possible compatible victims for each cell is recorded. The time variable was partitioned by considering periods of length duration $T$, starting out from the date of the event ${T}_{e}$ and going backward in time. The geographical variable was partitioned by considering areas of interest, associated with the relevant areas in the context of the historical phenomena under study. Then, each cell of the GeoTemporal partition will be associated with both, a geographical area and a specific range date of $T$ days of length. To simplify notation, cells with index numbers from 1 to $m$ are tagged. The referred cells look like Table 1.

**Table 1:** Example values of a table of ${N}_{j}$ (number of victims belonging to cell $j$) and ${I}_{j}$ (identified cases of cell $j$) for some $m=9$ GeoTemporal cells which result from using a particular temporal parameter $T=15$ days to define the length of the cells in temporal variable, and using the division of the region in areas of interest. Three areas are shown in this example. Column geo1 and row temp1 define the cell 1, column geo2 and row temp1 define the cell 2, and so on until cell $m$. In this example, $N=217$ and there are ${n}_{s}=22$ already-identified cases. In this example, listing the cells from 1 to 9 from left to right and from top to bottom, the prior probabilities for the cells are ${N}_{j}$/$N$ (0.161, 0.055, 0.014, 0.249, 0.106, 0.005, 0.258, 0.124, 0.028) and the posterior probabilities are the following values (0.013, 0.088, 0.001, 0.397, 0.134, 0.000, 0.188, 0.177, 0.002), which will be explained in the following subsection.

${N}_{j}$ (victims), ${I}_{j}$ (identified)	geo1	geo2	geo3	...
temp1	35, 0	12, 2	3, 0	...
temp2	54, 9	23, 3	1, 0	...
temp3	56, 4	27, 4	6, 0	...
...	...	...	...	...

#### Probabilities for every cell

Let us call ${{\mathscr{N}}}_{j}$ the subset of possible victims within $V$ which has the combination of variables associated with cell $j$, and let ${N}_{j}$ be the size of this set (${N}_{j}\,{\rm{=}}\,\#{{\mathscr{N}}}_{j}$). Thus, by $S\in {{\mathscr{N}}}_{j}$ we mean that UHR $S$ belongs to the set ${{\mathscr{N}}}_{j}$ (i.e., UHR $S$ corresponds to one of the individuals of subset ${{\mathscr{N}}}_{j}$). Let $P(S\in {{\mathscr{N}}}_{j})$ be the probability that $S$ correspond to an individual who belongs to cell $j$. In this way, $P(S\in {{\mathscr{N}}}_{j})$ is the prior probability that remains $S$ correspond to an individual who belongs to cell $j$, where *prior* refers to the probabilities calculated in an instance of knowledge before the data of the already-identified cases were observed; while $P(S\in {{\mathscr{N}}}_{j}| {\mathscr{D}})$ is the posterior probability that UHR $S$ come from cell $j$, where posterior refers to the instance of knowledge updated after the data of the already-identified cases were observed. Posterior probabilities will be obtained from prior probabilities after learning from the experience of the dataset of identified cases of the particular mass event of death (${\mathscr{D}}$). In the *Methods* section, it is described how to update mathematically prior probabilities $P(S\in {{\mathscr{N}}}_{j})$ into posterior probabilities $P(S\in {{\mathscr{N}}}_{j}| {\mathscr{D}})$ in a Bayesian treatment.

### Updating probabilities for every possible victim

Once the probability that remains $S$ belong to some victim of cell $j$ is updated (which in the sequel we denote by $P(S\ \in \ {{\mathscr{N}}}_{j}| {\mathscr{D}})=:{\widehat{\theta }}_{j}^{{\rm{post}}}$ for simplicity of notation), it is assumed that within that particular cell all the victims that are still unidentified (${{\mathscr{N}}}_{j}$) have the same chance of corresponding with UHR $S$. For this reason, the probability that remains $S$ correspond to the specific individual $i$ whose non-genetic variable values are associated with cell $j$ is:

2 $$P({v}_{i}\ is\ S| {\mathscr{D}})={P}_{j}^{i}=\frac{{{\widehat{\theta }}_{j}}^{{\rm{post}}}}{{N}_{j}}=\frac{1}{{N}_{j}}\left[\frac{\frac{{N}_{j}}{N}\left(\frac{N}{{N}_{k}}-2\right)+{I}_{j}}{\frac{N}{{N}_{k}}-2+{n}_{s}}\right],$$

where $${v}_{i}$$ is the $$i$$-th individual from cell $$j$$$$({v}_{i}\in \ {{\mathscr{N}}}_{j})$$, and $$k$$ is the cell in which there are more victims (see the section *Methods* below for details). The number of victims who are unidentified from cell $$j$$ is $${N}_{j}$$, and $${I}_{j}$$ is the total number of already-solved cases associated with cell $$j$$, such as $$\sum {I}_{j}={n}_{s}$$. The value of $${P}_{j}^{i}$$ will be the score associated with each individual $$i$$ of set $$V$$ belonging to cell $$j$$ to correspond with UHR $$S$$. The ranking of priorities for certain remains $$S$$ is built by listing the set of possible victims according to decreasing scores, which implies the existence of a ranking of blocks. Those victims, sharing the same score value (because they belong to the same cell), will be together in the same block.

### Validation techniques

The resulting scores are dependent on the particular partition of the subspace of non-genetic variables into cells. For each subset of non-genetic variables used, different partitions could be evaluated. The set of partitions will be used to group the victims into subsets of individuals. Each of these groupings will represent a “model”. Moreover, some variables, such as time, involve a way of partitioning that is in turn a function of a parameter that defines the length of the cell (parameter $T$) (for example, Table 1 shows example values of ${N}_{j}$ and ${I}_{j}$ for a particular GeoTemporal cells involving parameter $T=15$ days). Then, we propose the implementation of a sensitivity analysis of the results for the different partitions of the set of possible victims $V$ through cross-validation techniques, which provides information to identify the best subset of non-genetic variables to define the cells partition. It is noteworthy that the general methodology we propose in this paper is, in some sense, “*hybrid*”. That is because while we opted for a fully Bayesian treatment to update the matching probabilities, we chose a classical method for model selection via cross-validation. A fully Bayesian treatment would entail placing prior probabilities for different models (in this case partitions of the non-genetic variables) in the form of the so-called Bayes factors to combine the models in the final inference. Our choice of the hybrid method is due to two reasons: (i) there is no clear way to elicit priors for different models in our context; and (ii) using cross-validation for model selection is more amenable to be included automatically in computing code, without the need of interactive analysis by a statistical practitioner. This makes the method more attractive for applications by Forensic Scientists or professionals without specific statistical training. Broad applicability outside the specific statistical community is one of the main goals we seek in the dissemination of this work.

Cross-Validation divides the data of the already-solved cases randomly into two samples: (i) a learning sample ${{\mathscr{D}}}_{{\mathscr{L}}}$, in which several model options are comparatively estimated, aiming at the best fit; and (ii) a validation sample ${{\mathscr{D}}}_{V}$, used to evaluate the models with the reserved data. The cross-validation strategy is accepted in the literature as a way to prevent overfitting (i.e., proposing a model that works very well for the learning sample but very poorly with fresh data), while providing good predictions with new data^17^. The main idea is to implement the calculations described (and detailed in *Methods*) by using ${{\mathscr{D}}}_{L}$ instead of ${\mathscr{D}}$, to generate the results and evaluate the scores of those individuals who belong to subset ${{\mathscr{D}}}_{V}$ to quantify the results. It is necessary to define adequate magnitudes to measure the goodness of fit. Following Hastie^18^, ${{\mathscr{D}}}_{L}$ is selected from the original sample by taking a random statistical subset of $75$ percent size of the original sample from ${\mathscr{D}}$ (with no replacement); and the remaining cases form subset ${{\mathscr{D}}}_{V}$. The key is to pretend that the reserved cases have not been identified yet and to track and observe them in the final results.

In the ranking of possible victims for a particular learning sample ${{\mathscr{D}}}_{L}$, it is desirable i) that those victims belonging to the validation sample (${{\mathscr{D}}}_{V}$) obtain higher scores than those in the initial instance of knowledge and ii) that there are not many cases outside the validation sample that improve their scores. For those reasons, we focus on two magnitudes, i.e.:

- *Discriminating Power*, $DP$, which is defined as the fraction of the reserved cases which obtain greater scores than in the initial instance of knowledge (${{\mathscr{R}}}_{+}$) with respect to the size of the reserved sample (${\mathscr{R}}$, thus the size of set ${{\mathscr{D}}}_{V}$): $DP:={{\mathscr{R}}}_{+}/{\mathscr{R}}$. The idea here is that a useless model would have a low value of $DP$, similar to what would be obtained from setting a ranking of victims purely randomly. Such a model would not be useful at all for the validation sample, no matter how good it could have been for the learning sample.
- *Efficacy Rate*, $E$, which is defined as the ratio between the size of the reserved cases which improve their scores with respect to the initial instance of knowledge (${{\mathscr{R}}}_{+}$), and the total number of cases which improve their scores (${{\mathscr{N}}}_{+}$): $E={{\mathscr{R}}}_{+}$/${{\mathscr{N}}}_{+}$. Hence, heuristically, $E$ measures how *informative* the model, which is selected from the training sample, becomes for the validation sample.

Therefore, a good result achieves high values of both the *Discriminating Power* and *Efficacy Rate*. A cross-validation *realization* is defined as a particular division of sample ${\mathscr{D}}$ into two sub-samples: a so-called *learning sample* (denoted by ${{\mathscr{D}}}_{L}$) and an evaluating sample (denoted by ${{\mathscr{D}}}_{V}$). Several independent realizations (typically 50) are implemented for each partition of the non-genetic variables. Average of *Discriminating Power* and *Efficacy Rate*, <$DP$> and <$E$>, are calculated over all the realizations for each partition of the set of non-genetic variables.

Once the best set and partition of non-genetic variables to define cells has been chosen, the methodology is implemented to obtain ${P}_{j}^{i}$ of Eq. (2) using the complete sample ${\mathscr{D}}$ as a learning sample, since it is desirable to take the maximum advantage of the information provided by all the already-solved cases of the event under consideration.

**Figure 1:**
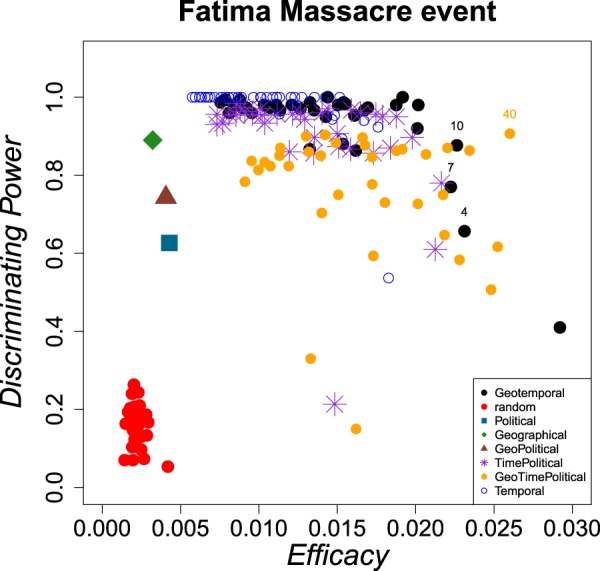
Discriminating Power $DP$ vs Efficacy Rate $E$ by considering different partitions of the space of variables for the Fátima event. Red dots represent the results obtained when the scores for individuals are randomly assigned to the sample. Black circles represent GeoTemporal cells; violet stars, TimePolitical cells; blue circles, Temporal cells; brown triangle, GeoPolitical cells; cyan square, Political cells; green triangle, Geographical cells, and orange circles, GeoTimePolitical cells. In all the partitions involving time variable, each symbol is associated with one temporal window to define the temporal length of the cell (from $T=1$ day to 90 days), in steps of 3 days.

Figure 1 shows average values of *Discriminating Power* and *Efficacy Rate* for different divisions of the space of non genetic variables into cells, obtained for the Fátima event, averaging $50$ independent cross-validation realizations, in each of which 25% of the cases were randomly selected as the validation sample ${{\mathscr{D}}}_{V}$. GeoTemporal partition of the space (black circles) shows the best results, both, in terms of Discriminating Power and Efficacy Rate. Different dots (black circles) correspond to different temporal windows to define cells, from 1 day to 90 days (in steps of 3 days, although in the figure only some of them are labeled). By considering only Temporal variables (empty blue circles) the Discriminating Power is maximum, but Efficacy Rate does not exceed a threshold value around 0.018. TimePolitical partition of the space (violet stars) shows similar results to the GeoTemporal one, although always a little worse. However, at the moment of choosing the best temporal parameter, there are results of GeoTemporal partition that are clearly better than the TimePolitical ones. The green diamond shows the results of using only Geographical variable; the blue square, only Political variable; and the brown triangle (which is approximately in the middle of Political and Geographical ones), using a partition into GeoPolitical cells. The Geographical partition achieves acceptable values of Discriminating Power, although very bad ones in terms of Efficacy Rate. Finally, orange circles represent results by considering the partition which combines the three types of variables, defining GeoTimePolitical cells. Clearly, by considering the three variables, the results are worse than considering only two (PoliticalTime, GeoPolitical and GeoTemporal ones). Red dots represent results when scores for individuals are randomly assigned; they make a well-separated cluster from the rest of the points. The fact that the GeoTemporal and TimePolitical variables lead to results much better than randomly assigned scores shows that there is a knowledge within identified cases of the Fátima event, and then it is possible to take advantage of this knowledge by learning about these non-genetic variables.

**Figure 2:**
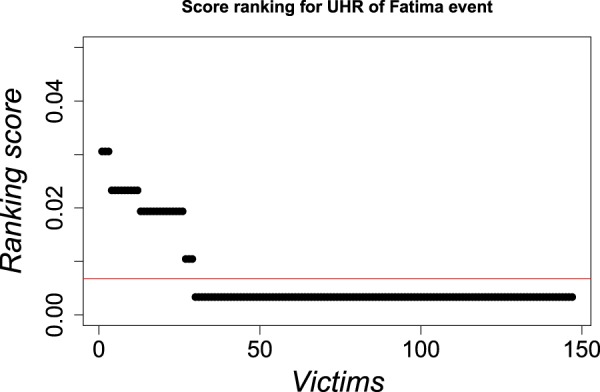
Example of a ranking score for UHR of a woman of (26,40) years old of Fátima event. Victims are represented on $x$-axes (as a sorted victim index), in decreasing order of the ranking scores. The continuous line (red) represents the value of the probability to correspond with certain UHR from Fátima at the initial instance of knowledge, before learning about the already-solved cases.

From results of Fig. 1, a temporal window of $T=10$ days using GeoTemporal variables was chosen to define the temporal length of Fátima event´s cells. Then, the methodology was implemented using the complete sample ${\mathscr{D}}$ as a learning sample, applying the expression (2) to obtain ${P}_{j}^{i}$ values of probability scores for every individual of the set of possible victims $V$ for the selected partition of non-genetic variables (which defines the ${N}_{i}$ and ${I}_{i}$ values for all $i$ cells, as the example of Table (1)). An example of ranking is shown in Fig. 2. In this Figure, the set of victims is represented on the $x$-axis; the chosen order in which individuals are represented is that of decreasing ranking scores. The continuous line (red) represents the value for the probability of corresponding with certain UHR from Fátima event at the initial instance of knowledge, before learning about the already-solved cases. Black points represent the probability for every victim in the updated instance of knowledge. In this piecewise-constant distribution, there are no possible victims with zero probability of corresponding with UHR from the Fátima event. This is a consequence of working within a Bayesian framework. The experience learned from the already-solved cases will not lead to establishing possible victims (with non-null probability) and not possible victims (with null probability), but will organize the possible ones hierarchically in terms of probability. Thus, all the possible victims remain in the complete set $V$ after updating the probabilities of the cells, until another type of assumption is made by which some of them are not possible anymore (as in the case of the sex of UHR is known).

**Figure 3:**
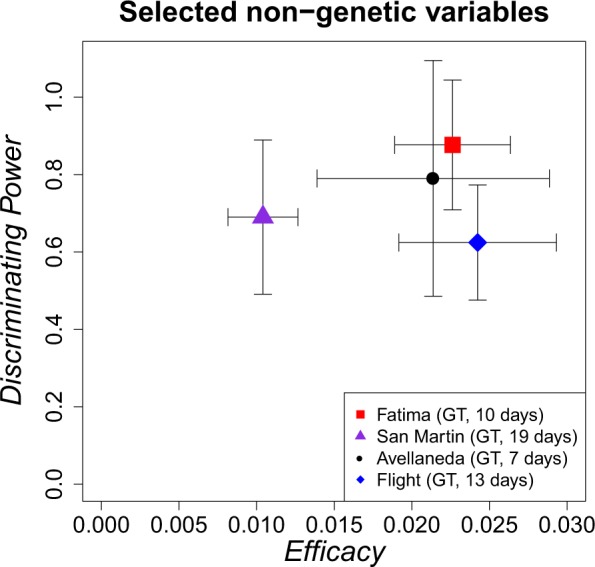
*Discriminating Power* $DP$ vs *Efficacy Rate* $E$ for the selected set of non-genetic variables for the different events under study (Fátima, San Martín, Avellaneda, and Flight events). In all the cases, the best partition is GeoTemporal cells (GT), although with a different temporal parameter to define the length of the cell, depending on the event.

Figure 3 shows the results for four mass events of the same context together: Fátima, San Martín, Avellaneda and a Flight event (which was one of the mechanisms applied in Argentina by some IDCs to get rid of people once their death had been decided). In the Figure, each point represents the results of Discriminating Power vs. Efficacy for the selected best partition of variables (GeoTemporal in all cases although with different values of temporal windows to define cells). Fátima event shows the best results (possibly as a consequence of the event characteristics) not only in terms of Discriminating Power and Efficacy Rate, but also in terms of small variance values. However, the results of Fátima overlap with the results of Avellaneda and Flight events in terms of Discriminating Power and Efficacy. Only results of San Martín event have less Efficacy Rate than the rest of the events, but in terms of Discriminating Power, it overlaps with the others too. Another important fact is that the GeoTemporal partition of the space of non-genetic variables is always the best way to detect patterns, although the best temporal windows (the temporal length of the cells) have different values for each event. Values are similar (from 10 to 20 days) possibly because the same phenomenon (the dictatorship in Argentina) underlies all the events.

## Discussion

For the UHR of a woman from the Avellaneda event, the methodology proposed the fourth position of the score rankings of victims for an individual for whom the EAAF only had a biological reference sample of a distant relative. After these results, the family was contacted to increase the number of reference samples from close relatives. Nowadays she is identified. This reflects the objective of this work: to build a tool that contributes to the work of forensic anthropologists regarding background information, taking advantage of the already-solved cases of a particular event.

The key of this work is very simple: turning to the already-solved cases of a mass event is essential to contribute to the knowledge regarding that event, knowledge that could be used in new searches of the same event, prioritizing the victims for certain UHR, and then, prioritizing the efforts to obtain new ante-mortem data and families’ blood samples within the identification process. Prioritization is essential in any investigation of a massive number of victims such as those involving crimes against humanity, conflicts, disaster victim identifications, among others.

## Methods

To understand how to update prior probabilities $P(S\in {{\mathscr{N}}}_{j})$ into posterior probabilities $P(S\in {{\mathscr{N}}}_{j}| {\mathscr{D}})$, it may be useful to think abstractly about a mathematically equivalent problem, which consists of throwing a die of $m$ faces. It is not possible to assure before collecting any data whether the die is fair (i.e., equal probabilities for every face) or loaded. The outcomes of a certain number of throws form the data ${\mathscr{D}}$. Then, the probabilities of each face will be inferred after the observation of a set of outcomes ${\mathscr{D}}$. In other words, the inference will capture how much unbalanced the die is according to experience. In our case, each face of this die represents each one of the cells of the problem under study. Following this analogy, $P(S\in {{\mathscr{N}}}_{j})$ is equivalent to the probability of obtaining face $j$ of the die in the next throw before observing the data ${\mathscr{D}}$ and $P(S\in {{\mathscr{N}}}_{j}| {\mathscr{D}})$ is the probability of obtaining face $j$ of the die in the next throw after learning about the data ${\mathscr{D}}$.

In the initial instance of knowledge, there is total uncertainty about which victim is associated with remains $S$, a fact which is consistent with assigning the same chance to each individual of $V$. As a consequence, $P(S\ \in \ {{\mathscr{N}}}_{j})={N}_{j}$/$N$. The sum of $P(S\ \in \ {{\mathscr{N}}}_{j})$ over all the cells $j$ is 1, following the assumptions (c) and (d), which means that with certainty UHR $S$ belongs to one of the possible victims of set $V$. To update $P(S\ \in {{\mathscr{N}}}_{j}| {\mathscr{D}})$ after learning about the identified cases, it is necessary to go back to the problem of the die, but imagining the experiment of throwing the $m$ faces die $n$ times, being $n$ the total number of already-solved cases in the problem. If face $i$ has a probability ${\theta }_{i}$ of resulting the winning face, it is possible to compute the probability of the outcome $({n}_{1},{n}_{2},\ldots ,{n}_{m})$ which means that face $1$ results ${n}_{1}$ times, and face $2$ results ${n}_{2}$ times and so on until the face $m$, which results ${n}_{m}$ times. The probability of obtaining the outcome $({n}_{1},{n}_{2},\ldots ,{n}_{m})$ is a well-known probability distribution called the Multinomial Distribution^19^:

3 $$P({n}_{1},{n}_{2},\ldots ,{n}_{m}|{\theta }_{1},{\theta }_{2},\ldots ,{\theta }_{m})=\frac{n!}{{n}_{1}!{n}_{2}!\,\ldots {n}_{m}!}{\theta }_{1}^{{n}_{1}}{\theta }_{2}^{{n}_{2}}\ldots {\theta }_{m}^{{n}_{m}}.$$

It is known that the mean value of the outcome ${n}_{1}$ is $E({n}_{1})=n{\theta }_{1}$, and in general $E({n}_{i})=n{\theta }_{i}$ for the face $i$. The multinomial distribution can be used to obtain the probability of a particular realization of an experiment in which it is possible to assume the probability of each outcome, or the existence of a model to generate the $n$ outputs for which outcome $1$ has probability ${\theta }_{1}$, outcome $2$, ${\theta }_{2}$, and so on up to outcome $m$, which has probability ${\theta }_{m}$. The problem under study is different empirically since there is (obviously) not a die involved, but human beings. Also, it is worth to remember that in Statistical Inference, instead of knowing the true parameters of the probability model, and using them to obtain probabilities of possible different samples, there is a single sample $({n}_{1},{n}_{2},\ldots ,{n}_{m})$ from which to learn the value of the parameters $({\theta }_{1},{\theta }_{2},\ldots ,{\theta }_{j})$. That single sample is the observed data ${\mathscr{D}}$ from the already-solved cases. In the context of this formalization of the problem, the data ${\mathscr{D}}$ of the already-solved cases can be thought as a particular realization of the $n$ throws of the die, which results in ${I}_{1}$ identified cases coming from cell $1$, ${I}_{2}$ from cell $2$, and so on up to ${I}_{m}$ identified cases from cell $m$. Thus, the data ${\mathscr{D}}$ became one possible result of the experiment of throwing the die, that is ${\mathscr{D}}=\{{I}_{1},{I}_{2},\ldots {I}_{m}\}$, where the sum of all the elements of ${\mathscr{D}}$ are ${n}_{s}$, which is the total number of identified cases: ${\sum }_{j=1}^{m}{I}_{j}={n}_{s}$.

It is worthwhile to note that thinking about the die as an abstract mathematical problem and the multinomial distribution that arises therein is actually an approximation. That is because a multinomial model for the data is akin to assuming sampling with replacement. In contrast, UHR recognition involves sampling without replacement, which would entail a multivariate hypergeometric distribution. Mathematically, opting for that approximation has the advantage of having a conjugate prior, which greatly simplifies calculations and makes available a posterior distribution and its first two moments as point estimators in closed-form. The alternative, i.e., the multivariate hypergeometric distribution, does not belong to the exponential family of distributions and thus it has no conjugate prior. Bayesian calculations for that model would entail numerical approximations for the arising integrals, or implementation of the Gibbs sampler, which would, in turn, require special computing code for the algorithms and convergence diagnostics. We aim our manuscript at Forensic researchers hoping they will implement the proposed methodology as a tool to prioritize cases and guide the searches. That simplicity in computational terms is the main reason why we adopt the multinomial approximation in this manuscript.

### Bayesian inference

Using the Bayes Theorem to update the probabilities in the ideal problem of the $m$-faced die, posterior probabilities of the parameters ${\theta }_{i}$ can be written in terms of prior probabilities:

4 $$f\left({\theta }_{1},{\theta }_{2}\ldots {\theta }_{m}| {\mathscr{D}}\right)=\frac{p\left({\mathscr{D}}| {\theta }_{1},{\theta }_{2}\ldots {\theta }_{m}\right)\ \cdot \ f\left({\theta }_{1},{\theta }_{2}\ldots {\theta }_{m}\right)}{P({\mathscr{D}})},$$

where

- $f\left({\theta }_{1},{\theta }_{2}\ldots {\theta }_{m}| {\mathscr{D}}\right)$ is the posterior density of the parameters $({\theta }_{1},{\theta }_{2}\ldots {\theta }_{m})$,
- $p\left({\mathscr{D}}| {\theta }_{1},{\theta }_{2}\ldots {\theta }_{m}\right)$ is the likelihood, which was expressed as a multinomial distribution in this problem,
- $f\left({\theta }_{1},{\theta }_{2}\ldots {\theta }_{m}\right)$ is the prior density of the parameters $({\theta }_{1},{\theta }_{2}\ldots {\theta }_{m})$,
- and the denominator $P({\mathscr{D}})=\int \cdots \int p\left({\mathscr{D}}| {\theta }_{1},{\theta }_{2}\ldots {\theta }_{m}\right)\cdot f\left({\theta }_{1},{\theta }_{2}\ldots {\theta }_{m}\right)\ d{\theta }_{1}\ldots d{\theta }_{m}$ is the integral (across the prior) of the product of the likelihood and the prior.

A Dirichlet distribution is considered as a prior density of the cell parameters $f\left({\theta }_{1},{\theta }_{2}\ldots {\theta }_{m}\right)$. By writing a prior distribution of the parameters, it is assumed that the parameters are not fixed values but random variables that have a certain probability distribution, which is expressed as a function of certain hyperparameters ${\alpha }_{1}$, ${\alpha }_{2}$, …, ${\alpha }_{m}$. It is given in Eq. (5):

5 $$f\left({\theta }_{1},{\theta }_{2},\ldots ,{\theta }_{m}\right)=\frac{\Gamma ({\alpha }_{1}+{\alpha }_{2}+\ldots +{\alpha }_{j}+\ldots )}{\Gamma ({\alpha }_{1})\Gamma ({\alpha }_{2})\ldots \Gamma ({\alpha }_{j})\ldots }{\theta }_{1}^{{\alpha }_{1}-1}{\theta }_{2}^{{\alpha }_{2}-1}\ldots {\theta }_{m}^{{\alpha }_{m}-1},$$

where each ${\theta }_{i}\ \ge \ 0$ and ${\theta }_{1}+\ldots +{\theta }_{m}=1$.

There are several reasons for which a Dirichlet distribution is used as a prior. The first one is that this distribution adapts to both informative and non-informative prior distributions since it allows the calibration of the hyperparameters for both situations. *Informative* means that not all the possible outcomes are equally likely to occur (as in our case). The second reason is that the Dirichlet is the conjugate prior for the Multinomial distribution, which means that the following property is met: if the likelihood of the parameters is a Multinomial distribution and the prior density of the parameters is a Dirichlet one, then the Posterior density of the parameters $f\left({\theta }_{1},{\theta }_{2}\ldots {\theta }_{m}| {\mathscr{D}}\right)$ is also a Dirichlet distribution but with updated values of the parameters, which are going to be a known function of the hyperparameters and the data ${\mathscr{D}}$.

By having a distribution of probability associated, the parameters $\theta$ have an associated uncertainty. The following expressions are met for the expected values and variance of the parameters ${\theta }_{j}$ ($E({\theta }_{j})$ and $Var({\theta }_{j})$ respectively) as a function of the hyperparameters ${\alpha }_{j}$ for all $j$ value:

6 $$E({\theta }_{j})=\frac{{\alpha }_{j}}{{\alpha }_{0}}\ \ \ \ Var({\theta }_{j})=\frac{{\alpha }_{j}({\alpha }_{0}-{\alpha }_{j})}{{\alpha }_{0}^{2}({\alpha }_{0}+1)},$$

where ${\alpha }_{0}={\alpha }_{1}+{\alpha }_{2}+\ldots +{\alpha }_{m}$. Those properties render a very amenable interpretation for prior elicitation (*i.e*. translating expert knowledge into concrete values)^16^. GeoTemporal cell probabilities at the initial instance of knowledge of the problem have to be consistent with what is known before the already-solved cases. This means that all the possible victims are equally probable, and therefore those cells with more victims will be more likely. As a consequence, before the data from the identifications, the prior probabilities of the cells are proportional to the number of victims belonging to every GeoTemporal cell. A further condition is that the parameters of the distribution are such that the prior expectation of ${\theta }_{j}$, $E({\theta }_{j})={N}_{j}/N$ for all $j=1,2\ldots ,m$. In this way, it is required that all individuals have the same chance of corresponding with UHR $S$ in the instance of the knowledge prior to the data of the already-solved cases. As for the Dirichlet distribution it is known that the expected value of the variable ${\theta }_{j}$ is ${\alpha }_{j}$/${\alpha }_{0}$, from Eq. (6), then what must be satisfied are $m$ conditions, one for each $j$ cell:

7 $$E({\theta }_{j})=\frac{{\alpha }_{j}}{{\alpha }_{0}}=\frac{{N}_{j}}{N}.$$

The $m$ conditions of Eq. (7) are not independent, since the sum of ${\alpha }_{j}$ over all $j$ from $1$ to $m$ is ${\alpha }_{0}$. This fact implies that it is not possible to solve the system equations of $m$ unknown variables and only $m-1$ independent equations. It is necessary to propose another condition as an extra equation. The coefficient of variation ($CV$) of a random variable is defined as the quotient between its standard deviation and its expected value. As a conventionally accepted “rule of thumb”, random variables with a  coefficient of variation greater than 0.5 are considered very heterogeneous, while those with values lower than 0.10 are considered very homogeneous. Since it is expected to specify a fairly vague prior, it is possible to establish the coefficient of variation of the most populated cell to be 1, as a criterion to establish the needed extra condition, *i.e*.,

8 $$\,{\rm{CV}}\,({\theta }_{k})=\frac{\sqrt{Var({\theta }_{k})}}{E({\theta }_{k})}=1,$$

where cell $k$ is that cell for which ${N}_{k}$ is maximum over all the ${N}_{j}$ cell values. The fact that this condition is met for the most populated cell ensures that the coefficient of variation will be more than $\,{\rm{1}}\,$ for the rest of the cells. By squaring the expression of Eq. (8) and replacing it in the second one of Eq. (6), the following extra condition for the hyperparameters is obtained:

9 $$\frac{{\alpha }_{k}({\alpha }_{0}-{\alpha }_{k})}{{\alpha }_{0}^{2}({\alpha }_{0}+1)}={\left(\frac{{\alpha }_{k}}{{\alpha }_{0}}\right)}^{2}$$

After solving the system of equations given by the Eqs. (7) and (9), an expression for ${\alpha }_{0}$ as a function of the data is obtained as: ${\alpha }_{0}=\frac{N}{{N}_{k}}-2$. By replacing this result in Eq. (7), an expression for every hyperparameter ${\alpha }_{j}$ corresponding to cell $j$ is obtained:

10 $${\alpha }_{j}=\frac{{N}_{j}}{N}\left(\frac{N}{{N}_{k}}-2\right)\ \ \ \ \forall \ \ j$$

It is important to note that if ${N}_{j}=0$ for cell $j$, then ${\alpha }_{j}=0$, which implies that the expected value is zero $\left(E({\theta }_{j})=0\right)$ for this cell. In other words, if there are no cases of kidnapped individuals within the place and dates corresponding with the GeoTemporal cell $j$, then the probability of remains $S$ to be associated to somebody from this cell is null, which makes sense because there are no people with that combination of values of the variables.

Using the property of conjugate distributions for the Dirichlet (prior distribution) and Multinomial (likelihood), the posterior distribution results in a Dirichlet distribution with updated parameters:

11 $$f\left(\theta ,{\theta }_{2},\,\ldots \,,{\theta }_{m}\right)=\frac{\Gamma ({\alpha }_{1}^{{\prime} }+{\alpha }_{2}^{{\prime} }+\ldots +{\alpha }_{j}^{{\prime} }+\ldots )}{\Gamma ({\alpha }_{1}^{{\prime} })\Gamma ({\alpha }_{2}^{{\prime} })\ldots \Gamma ({\alpha }_{j}^{{\prime} })\ldots }{\theta }_{1}^{{\alpha }_{1}^{{\prime} }-1}{\theta }_{2}^{{\alpha }_{2}^{{\prime} }-1}\ldots {\theta }_{m}^{{\alpha }_{m}^{{\prime} }-1}$$

where values of ${\alpha }_{1}^{{\prime} }$, ${\alpha }_{2}^{{\prime} }$, …, ${\alpha }_{m}^{{\prime} }$ are functions of the hyperparameters $\alpha$ and the data ${\mathscr{D}}$, as: ${\alpha }_{j}^{{\prime} }={\alpha }_{j}+{I}_{j}$, in general for cell $j$. This means that the expected values of the posterior parameters ${\theta }_{1}$, ${\theta }_{2}$, …, ${\theta }_{m}$ are:

$${E}^{post}({\theta }_{j})=\frac{{\alpha }_{j}^{{\prime} }}{{\alpha }_{0}^{{\prime} }}=\frac{{\alpha }_{j}+{I}_{j}}{{\alpha }_{0}+{n}_{s}}$$

These are the Bayesian estimators of the unknown probabilities which use both the knowledge given by the prior distribution (equiprobability of all the eligible victims) and the observed data (the set of already-solved cases) for a particular cell partition of non-genetic variables. This means that in the problem of the die the probability that the outcome is face $j$ (the cell $j$ in the problem at hand) will depend on the prior expected value modified by the data ${\mathscr{D}}$ from the already-solved cases (both of the total already-solved cases identified ${n}_{s}$ and of the total of already-solved cases that fell into that cell, ${I}_{j}$). These results imply that the probability that remains $S$ belong to some victim of cell $j$, which is $P(S\ \in \ {{\mathscr{N}}}_{j}| {\mathscr{D}})$ (called ${\theta }_{j}^{post}$ for the sake of simplicity), is:

$$P(S\ \in \ {{\mathscr{N}}}_{j}| {\mathscr{D}})=\frac{{\alpha }_{j}+{I}_{j}}{{\alpha }_{0}+{n}_{s}}=\frac{\frac{{N}_{j}}{N}\left(\frac{N}{{N}_{k}}-2\right)+{I}_{j}}{\frac{N}{{N}_{k}}-2+{n}_{s}}\equiv {\widehat{{\theta }_{j}}}^{{\rm{post}}}$$

This work was accepted and presented in the Congress of the American Academy of Forensic Sciences^20^. Data are not available but all the scripts for implementing the methodology in R are available in https://github.com/inescaridi/PriorID as the project *priorID*. An open, free, multi-platform and standalone interface for users to implement this methodology in diverse problems and incorporate feedback from forensic researchers is now under construction.
